# Weak Degradation Characteristics Analysis of UAV Motors Based on Laplacian Eigenmaps and Variational Mode Decomposition

**DOI:** 10.3390/s19030524

**Published:** 2019-01-27

**Authors:** Xiaohong Wang, Wenhui Fan, Xinjun Li, Lizhi Wang

**Affiliations:** 1 School of Reliability and Systems Engineering, Beihang University, Beijing 100191, China; wxhong@buaa.edu.cn; 2 School of Mechanical Engineering and Automation, Beihang University, Beijing 100191, China; fanwenhui@buaa.edu.cn (W.F.); lixinjun@buaa.edu.cn (X.L.); 3 Institute of Unmanned System, Beihang University, Beijing 100191, China; 4 Key Laboratory of Advanced Technology of Intelligent Unmanned Flight System of Ministry of Industry and Information Technology, Beihang University, Beijing 100191, China

**Keywords:** variational mode decomposition, Laplacian eigenmaps, multi-rotor unmanned aerial vehicle, brushless direct current motor, weak degradation characteristics

## Abstract

Brushless direct current (BLDC) motors are the source of flight power during the operation of rotary-wing unmanned aerial vehicles (UAVs), and their working state directly affects the safety of the whole system. To predict and avoid motor faults, it is necessary to accurately understand the health degradation process of the motor before any fault occurs. However, in actual working conditions, due to the aerodynamic environmental conditions of the aircraft flight, the background noise components of the vibration signals characterizing the running state of the motor are complex and severely coupled, making it difficult for the weak degradation characteristics to be clearly reflected. To address these problems, a weak degradation characteristic extraction method based on variational mode decomposition (VMD) and Laplacian Eigenmaps (LE) was proposed in this study to precisely identify the degradation information in system health data, avoid the loss of critical information and the interference of redundant information, and to optimize the description of a motor’s degradation process despite the presence of complex background noise. A validation experiment was conducted on a specific type of motor under operation with load, to obtain the degradation characteristics of multiple types of vibration signals, and to test the proposed method. The results proved that this method can improve the stability and accuracy of predicting motor health, thereby helping to predict the degradation state and to optimize the maintenance strategies.

## 1. Introduction

Recently, multi-rotor unmanned aerial vehicles (UAVs) have been widely used in many fields such as inspection, mapping, and rescue, due to their excellent control performance and attitude stability. The flight status and mission safety of UAVs are of great importance in the fields of agricultural production, rescue and disaster relief, and military operations [1,2]. The brushless direct current (BLDC) motor is a critical component of the power system of multi-rotor UAVs, so that its working state directly affects the success rate and safety of task execution. Although the rotary-wing UAV usually has multiple rotors to ensure its flight conditions, the fault of a single rotor or the motor can have devastating consequences for the flight mission of the UAV. A multi-rotor UAV is a typical system with high mission safety requirements, and so conservative maintenance strategies are often preferred over the life of the vehicle, but if the motor is replaced long before it reaches its service life, the cost will be greatly increased. However, if the use time is extended without knowing the lifespan of the motor, the flight safety of the UAV cannot be guaranteed. Therefore, estimating the working state of the BLDC motor can help to adjust the maintenance and replacement strategy of the UAV and reduce the flight cost.

In the current stage, fault diagnosis and health management technology for rotating parts, such as motors, has been widely applied in industry. It is a very effective method to obtain information about the state of the rotating parts, based on vibration signals. In recent years, there are a large number of researchers studying novel approaches for rotating machinery, such as Feng [3] and Qu [4]. Some researchers try to apply advanced methods on vibration signal processing [5,6]. Thus, it can be seen that vibration signals are still one of the most common indicators of the health status of rotating machinery. In general, damage faults will show obvious periodic pulse signals during the rotating process, and will clearly recognizable impact vibration can be found on the vibration signal spectrum, which is conducive to identification and effective classification [7,8]. Liu et al. utilized energy entropy to detect the chatter occasions [9]. Mohanty et al. analyzed vibro-acoustic features of the bearing for diagnosis [10]. These are typical examples of damage fault detection by the features of the vibration signals. However, abrasion faults undergo gradual changes with the increase of the use time. Since its vibration properties are characterized by strong similarity, weak distinction, and strong randomness, it is a typical weak fault. It is hard to identify weak fault characteristics with the traditional fault diagnosis method, so that the extraction of weak fault characteristics is a difficult task in the health management of mechanical parts. A favorable data processing method is indispensable for achieving the effective identification of fault information. Some studies have been carried out on the extraction of weak degradation characteristics of vibration signals. Hou et al. used global optimization sparse coding and the approximate SVD (singular value decomposition) method to study weak fault characteristics extraction of rolling bearings [11]. Based on multipoint kurtosis and VMD (variational mode decomposition), Cai et al. improved the EMD (empirical mode decomposition) and EEMD (ensemble empirical mode decomposition) methods for the extraction of weak fault characteristics of bearings [12]. Yu et al. proposed a method based on SCS (sparse coding shrinkage) in ITD (intrinsic time-scale decomposition) for weak fault characteristics extraction of bearings [13]. Li et al. carried out researches on the weak fault detection of rotating machinery based on the ADMM (alternating direction method of multipliers) [14] and I-NCRA (improved non-convex regularization algorithm) [15]. The existing research covers most of the excellent signal processing method, but there are still imperfection. Some researchers focus on the improvement of the signal analysis approach, including but not limited to current [16] and vibration signals, such as Hou and Cai et al., so they do not consider helping with the direct prediction or estimation of the health status. Besides that, some researchers work on the fault detection of the rotating machinery, who only pay attention to the fault occasions without considering the development process analysis.

Nevertheless, the cost-saving efficiency brought about by preventive maintenance within the life cycle of equipment is much higher than that of corrective maintenance [17,18]. Currently available studies on the extraction of weak fault signals mainly focus on the identification of components with sudden changes, such as pulse components, without paying attention to the fault development processes of mechanical parts in the early stage, so that the literature cannot effectively guide the preventive maintenance of faults. To realize fault forecasting, and to take appropriate preventive maintenance measures, it is essential to analyze the development process of the fault in the early stage. However, for an aircraft with an aerodynamic structure such as the multi-rotor UAV, the vibration environment of the onboard equipment is more unstable than that of the ground equipment, and the noise component is complicated. Consequently, the effectiveness of the traditional method to filter the interference components by setting a separation standard has been greatly reduced, making it difficult to effectively extract the weak degradation process in the early stage of the fault. To overcome this defect, instead of distinguishing the data signals by the frequency component, the manifold learning method extracts the nonlinear essential relationship of the characteristics data from the perspective of the data point distance, which can successfully solve the problem that it is hard to distinguish useful information from noise while processing frequency–domain signals. For example, Xu et al. used the Isomap method to analyze multi-type sensitive characteristics of rotating faults [19]. Chen et al. used LE (Laplacian Eigenmaps) and the DT-CWT (dual-tree complex wavelet transform) method to study the method of identifying gear faults, so as to solve the problem of excessive redundant information in the vibration signal [20]. These are attempts to utilize the manifold method for vibration signal processing. Besides that, Shao et al. also adopted the locally linear embedding method to monitor the performance degradation of rolling bearings [21]. However, in their research, only limited characteristics are considered, leading to the lack of information completeness. Therefore, the health status information in weak signals cannot be captured adequately for accuracy prediction and estimation. Therefore, to extract weak degradation characteristics in the early stage of the fault, such as data-driven information analysis methods and data dimension reduction methods can help solve the problem that complex background noises cannot be separated, and weak degradation information is difficult to obtain.

In view of the difficulty of effectively identifying the development trend of weak degradation characteristics during the abrasion fault of the BLDC motor for multi-rotor UAVs, the aim of this study was to overcome the poor performance of the traditional signal analysis method in information separation when there are many noise components. The proposed method used the VMD signal separation method and the LE method to extract and fuse the multi-characteristics parameter information based on the variation trend of the abrasion fault with the increase of time. The progressive abrasion fault development process of the motor was identified to monitor the health status of the motor, and predict the moment of the motor fault. Finally, an experiment was designed to collect data about the actual working conditions of the multi-rotor UAV motor, to verify the effectiveness of the method proposed in this study.

The structure of this paper is as follows: In the second section, the basic principles of the methods used in the paper are briefly introduced, based on which the main ideas of the technical method proposed in this study are presented. In the third section, an experiment on the multi-rotor UAV motor is described, and the method and key points of collecting the vibration data of the multi-rotor UAV motor are introduced. In the fourth section, the results are analyzed and explained in detail, and the conclusions are given. In the fifth section, the study is summarized.

## 2. Methods

In general, in the process of characteristics analysis of vibration signals of mechanical parts, the time-domain signals are denoised in advance to avoid the negative impacts of noise. However, in the degradation process of the multi-rotor UAV motor, the gradual-change characteristics of the abrasion fault have weak separable characteristics in the strong background noise, whereas it is much easier for the characteristics components of damage fault to stand out among time-domain signals. As a result, the traditional denoising process will greatly weaken the gradual-change characteristics of the abrasion fault. To solve this problem, the traditional mode of carrying out frequency-domain denoising before characteristics extraction was abandoned in this study; instead, the time series vibration characteristics rather than the original vibration signals were used as the basic input for the analysis and extraction of degradation information, based on the idea of multi-dimensional characteristics fusion.

**Figure 1:**
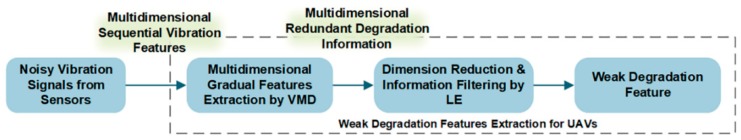
Principle and flowchart of the proposed method.

Thus, the following method (as shown in Figure 1) was used to process the signals in the actual working condition to preserve the sufficient abrasion fault evolution characteristics. Firstly, on the basis of completely preserving the original time-domain vibration signals (signals with noises), multi-type time series characteristics of the signals were extracted to ensure that the weak gradual-change characteristics of the abrasion fault would not be lost. Secondly, the VMD method was applied to process fluctuant noises among multi-type timing series signals and to extract the trend information in time series vibration characteristics for fusing multi-dimensional characteristics. Finally, the LE method was used to fuse the multi-dimensional time series characteristics. This step was essentially to extract information valuable for the trend signals and further filter repeated information and noises for the judgment and prediction of the running state. This method is able to avoid the loss of trend information in the denoising process of the time-domain signals, and to remove the non-trend noises from the time series characteristic signals on the premise of sufficiently retaining the vibration signals. Thereby, the proposed method achieves the purpose of accurately extracting and retaining the weak degradation characteristics.

### 2.1. Charicteristics of the Vibration Signal

The BLDC motor is a typical rotating machine, and mature research on the characterization of its vibration signals can be found in the literature. Generally speaking, characteristics of vibration signals are divided into frequency-domain characteristics and time-domain characteristics. Time-domain characteristics describe the statistical characteristics of the vibration signals in the domain of time, and the commonly used indexes include mean value, variance, root-mean-square value, peak, skewness, kurtosis, waveform, pulse, and margin. Frequency-domain characteristics describe the frequency-domain components of the vibration signals based on the Fourier transform. In this study, gravity frequency, mean square frequency, and frequency variance were used to describe changes in the gravity position of the spectrum and changes in energy distribution. To enhance the description of the overall frequency-domain characteristics of the signals, and to compensate for the time-invariant defects of the Fourier transform-based frequency domain analysis method in describing the non-stationary signals, frequency domain analysis, and the mean value and entropy of the Hilbert marginal energy spectrum based on the Hilbert Huang Transform (HHT) time were used as supplementary characteristics for comprehensive analysis in this study. For the time-domain signal x(t), its Hilbert marginal energy spectrum was defined as [22]:

(1) $$E(f)=\int_{0}^{T}H^{2}\left(f,t\right)dt,$$

where H(f,t) is the Hilbert spectrum of signal x(t) obtained by the HHT method. Therefore, the mean value of the Hilbert marginal energy spectrum can be defined as:

(2) $$S=\sum_{k}\left[E(f_{k})\right],$$

According to the theory of information [23], the entropy of its Hilbert marginal energy spectrum was defined as:

(3) $$\hat{H}=-\sum_{j=1}^{m}p_{j}\log_{2}p_{j},$$

where $p_{j}=E(j)/\sum_{j=1}^{m}E(j)$, m is the number of frequency components, and $\sum_{j=1}^{m}p_{j}=1$.

All indexes above are indexes of characteristics of vibration signals commonly used in the health monitoring of rotating machinery. In this study, the dimension reduction of characteristics of vibration signals was carried out based on the above-mentioned characteristic indexes.

### 2.2. Variational Mode Decomposition

Based on the parameters of the characteristics of multi-type vibration signals described in the previous section, the VMD method was used to pre-process the time series degradation characteristics of the tested motor. The pre-processing mainly aimed to remove the noise and abnormal fluctuations in the time series degradation characteristics, and to retain the time series variation trend of the abrasion fault. The VMD algorithm was proposed by Dragomiretskiy and Zosso in 2014 [24]. Unlike the commonly used EMD and LMD (local mean decomposition) algorithms, the VMD algorithm applied non-recursive mode decomposition, which could simultaneously estimate the modalities of different center frequencies. The basis of the VMD algorithm is a set of adaptive Wiener filters, which not only avoided the constant accumulation of envelope estimation errors, but also overcame the end effect [25].

The decomposition process of the VMD algorithm is essentially the process of solving the variational problem. Covering the construction and solution of the variational problem, this algorithm mainly involves three important concepts: classical Wiener filtering, Hilbert transform, and frequency mixing [26]. VMD algorithm consists of two steps:

● Step 1: Construction of the variational problem

In the construction of the variational problem, each “mode” was assumed to be a finite bandwidth with a center frequency, and then the variational problem could be described as seeking *k* modal functions $u_{k}\left(t\right)$. To minimize the sum of the estimated bandwidths of each mode, the constraint condition is that the sum of each mode should be the original input signal f. The details are explained below:

1. Obtain the analytic signal of each mode function $u_{k}\left(t\right)$ by the Hilbert transform, aiming at capturing the one-sided spectrum $\left(\delta \left(t\right)+\frac{j}{\pi t}\right)*u_{k}\left(t\right)$.
2. Add an estimation center frequency item $e^{j\omega_{k}t}$ to each analytic signal, so that the frequency spectrum of each mode is modulated to corresponding baseband $\left[\left(\delta \left(t\right)+\frac{j}{\pi t}\right)*u_{k}\left(t\right)\right]e^{-j\omega_{k}t}$.
3. Then, calculate the L2 norm of the above signal, and estimate the bandwidth of each mode signal. The variational problem can be described as:
  (4) $$\underset{\left\{u_{k}\},\{\omega_{k}\right\}}{\min}\left\{{\sum_{k}\left\|\partial_{t}\left[\left(\delta \left(t\right)+\frac{j}{\pi t}\right)*u_{k}\left(t\right)\right]e^{-j\omega_{k}t}\right\|}^{2}\right\}\;s.t.\sum_{k}u_{k}=f,$$
  where $\left\{u_{k}\right\}:=\left\{u_{1},\cdots ,u_{k}\right\}$, $\left\{\omega_{k}\right\}:=\left\{\omega_{1},\cdots ,\omega_{k}\right\}$, $\sum_{k}:=\sum_{k-1}^{k}$.

● Step 2: Solution of the variational problem

1. On the above basis, the quadratic penalty factor, α, and the Lagrangian multiplication operator, λ(t), were introduced, and the constrained variational problem was transformed into an unconstrained variational problem to obtain the extended Lagrangian expression. The extended Largangian expression is expressed as:
  (5) $$L\left(\left\{u_{k}\right\},\left\{\omega_{k}\right\},\lambda \right):=\alpha \sum_{k}{\left\|\partial_{t}\left[\left(\delta \left(t\right)+\frac{j}{\pi t}\right)*u_{k}\left(t\right)\right]e^{-j\omega_{k}t}\right\|}^{2}+{\left\|f\left(t\right)-\sum_{k}u_{k}\left(t\right)\right\|}_{2}^{2}+\langle \lambda \left(t\right),f\left(t\right)-\sum_{k}u_{k}\left(t\right)\rangle ,$$
2. Next, the ADMM was used to solve the above variational problem, and the “saddle point” of extending the Lagrangian expression was worked out by alternately updating $u_{k}^{n+1}$, $\omega_{k}^{n+1}$, and $\lambda^{n+1}$ so as to solve $\left\{u_{k}(t)\right\}$, the set of k modal functions under constraint conditions. The specific solution algorithm is not expounded in this paper. Refer to [24] for more details. Thus, that we can obtain mode functions of the original signal set $\left\{u_{k}(t)\right\}$. As the independent frequency-domain component of the original input signals, this set efficiently separated different frequency-domain components in the original signals.

**Figure 2:**
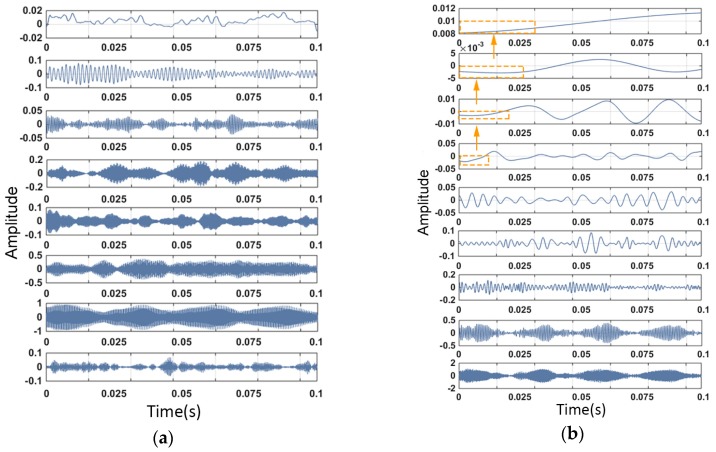
Comparison of time-domain signals of (**a**) the VMD (variational mode decomposition) method and (**b**) the EMD (empirical mode decomposition) method.

**Figure 3:**
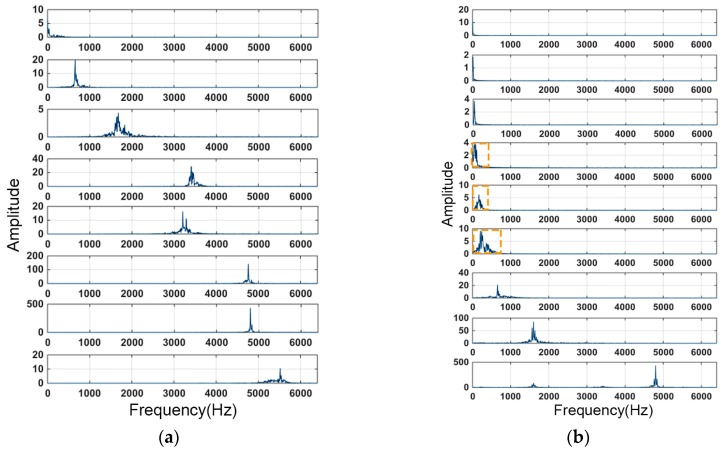
Comparison of frequency–domain signals of (**a**) the VMD method and (**b**) the EMD method.

The VMD and the traditional EMD method were used to decompose and compare the time-domain signals of the BLDC motor with load. The comparison of the time-domain components and their corresponding frequency-domain components is shown in Figure 2 and Figure 3.

Figure 2 demonstrates the time-domain mode signals decomposed by VMD and EMD from the same vibration signal sample. Each graphic in Figure 2 represents one mode of the sample, which can also be represented as $u_{k}\left(t\right)$. The corresponding frequency-domain signal of $u_{k}\left(t\right)$ is revealed in Figure 3. It can be clearly seen from Figure 2 that the EMD method had a significant end effect, and during the recursive process from high frequency to low frequency, the oscillation of the end-point data was continuously polluted inward. As shown in Figure 3, in this case, the EMD method exhibited modal aliasing of multiple frequency-domain components, unlike the VMD method, and the components of each decomposition component failed to be clearly distinguished. The VMD method had a clear advantage in frequency separation.

### 2.3. Laplacian Eigenmaps

The basic idea of the LE method was to describe a manifold through an undirected weighted graph and then to find a low-dimensional representation by embedding the graph; that is, to redraw the manifold in the high-dimensional space in a low-dimensional space on the premise of maintaining the local adjacency of the data [27]. The basic principle of the LE method was to ensure that the adjacent points in the high-dimensional manifold were still adjacent in the low-dimensional manifold, and the smaller the neighborhood, the more clear the linear structure. However, it was necessary to ensure sufficient overlap between the neighborhoods so that there could be enough connections between the distant points.

In fact, the LE method constructed the relationships between data points from the perspective of the graph, and the data was assumed to have local structural properties. Each data point was regarded as a vertex of the graph, and each edge between two data points had a corresponding weight representing the similarity between the two points. The more similar the two data points, the larger the weight of the edge. The LE method assumed that each point was only similar to those points closest to them, and the similarity between data points farther away from each other was zero, so that the points after dimension reduction were kept as close as possible.

The purpose of the LE method is to map this weighted graph to a line so that a minimum spacing can be maintained between the adjacent points. For this problem, the specific implementation method is as follows:

● Step 1: Manifold Construction

Let $x={\left(x_{1},x_{2},\cdots ,x_{N}\right)}^{T}$ and $y={\left(y_{1},y_{2},\cdots ,y_{N}\right)}^{T}$, where $x_{i},y_{i}\in \mathbb{R}$ is the coordinate value of the *i*th point in $M^{d}$ and ${\mathbb{R}}^{m}$, and this problem can be transformed into finding $y_{i}\in \mathbb{R}$ to minimize $\sum_{i,j}{\left(y_{i}-y_{j}\right)}^{2}W_{ij}$ under reasonable constraints. For any value of y, $\frac{1}{2}\sum_{i,j}{\left(y_{i}-y_{j}\right)}^{2}W_{ij}=y^{T}Ly$. The Laplacian matrix is L=G−W, where G is the degree matrix of the graph, and W is the adjacency matrix. It is not difficult to find that $W_{ij}$ is a symmetric matrix and $G_{ii}=\sum_{j}W_{ij}$, from which the following equation can be derived:

(6) $$\begin{array}{ll} \sum_{i,j}{\left(y_{i}-y_{j}\right)}^{2}W_{ij} & =\sum_{i,j}\left(y_{i}^{2}+y_{j}^{2}-2y_{i}y_{j}\right)W_{ij}\\ & =\sum y_{i}^{2}D_{ii}+\sum y_{j}^{2}D_{jj}-2\sum y_{i}y_{j}W_{ij}\\ & =2y^{T}Ly, \end{array}$$

● Step 2: Solution of the constrained optimization problem

Thus, the problem of solving the minimum value can be transformed into seeking $\arg\min_{y^{T}Dy=1}y^{T}Ly$. It can be found from Equation (4) that *L* is a positive semi-definite matrix, and the vector that minimizes the objective function was given by the minimum eigenvalue of the generalized eigenvector problem, Ly=λGy. Hence, the problem and its constraints were described as $y_{opt}=\arg\min_{\begin{array}{l} y^{T}Dy=1\\ y^{T}D1=0 \end{array}}\;y^{T}Ly$. In summary, the mapping of this graph was given by $Y=\left[y_{1}y_{2}\cdots y_{d}\right]$, the matrix of N×d, where $Y_{i}^{T}$ in the *i*th row provides the embedding coordinates of the *i*th vertex. Likewise, the problem of minimizing $\sum_{i,j}{\left\|Y_{i}-Y_{j}\right\|}^{2}W_{ij}=tr\left(Y^{T}LY\right)$ was simplified to finding $Y_{opt}=\arg\min_{Y^{T}DY=1}tr\left(Y^{T}LY\right)$. According to the low-dimensional manifold of the *N* data points, $y={\left(y_{1},y_{2},\cdots ,y_{N}\right)}^{T}$ is the manifold mapping of $x={\left(x_{1},x_{2},\cdots ,x_{N}\right)}^{T}$ in the low-dimensional space.

### 2.4. Degradation Characteristics Extraction Based on VMD and LE

**Figure 4:**
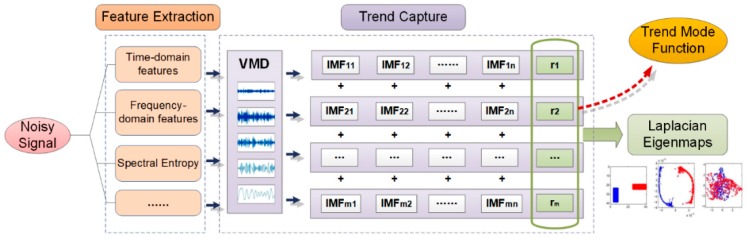
Proposed method for the degradation feature extraction.

Based on the VMD method and the LE manifold dimension reduction theory introduced in the previous section, a state evolution trend analysis method was proposed, based on the extraction and fusion of the multi-dimensional vibration characteristics’ degradation trends (as shown in Figure 4).

● Step 1: Extraction of original characteristics

For a degraded component, the time series sample of vibration signals within its full life cycle T was found to be $s={\left(s_{1}(t),s_{2}(t),\cdots ,s_{n}(t),\cdots ,s_{N}(t)\right)}^{T}$, where *N* is the number of time series samples. For the time series sample $s_{n}(t)$, its characteristic set was defined as $F_{n}=\{f_{n,d}|d\in [1,D]\}$, where D is the characteristic dimension. Then, the time series of the characteristics in the d dimension was obtained, which is $c_{d}(n)={\left(f_{1},f_{2},\cdots ,f_{N}\right)}^{T}$. Hence, a characteristics matrix was formed, that is, $F=(c_{1},c_{2,},\cdots ,c_{D})$.

● Step 2: Construction of a high dimensional manifold

According to the basic theory of the VMD method, the variational problem of each characteristic time series, $c_{d}(n)={\left(f_{1},f_{2},\cdots ,f_{N}\right)}^{T}$ was constructed and solved to seek its modal function set, $U_{d}=\{u_{k}^{d}\left(n\right)|k\in N^{+}\}$, which satisfies $c_{d}(n)=\sum_{i=1}^{k}u_{k}^{d}(n)$. The appropriate trend term in the modal function set was selected as the information output of the evolution trend of each characteristic. Subsequently, the evolution trend set $U_{tr}=\{u_{r}^{d}(n)|d=(1,2,\cdots ,D)\}$ was obtained for each characteristic in the D dimension. Then, a high-dimensional manifold, $M^{D}$, containing N data points and existing in the D dimension was constructed.

● Step3: Manifold dimension reduction

The coordinates of *N* time series data points were defined as $x={\left(x_{1},x_{2},\cdots ,x_{N}\right)}^{T}$. If a random point $A\in M^{d}$ has k neighboring points, then a weighted graph G=(V,E) with the number of nodes being k can be constructed for each point and its neighbors. The manifold dimension reduction problem can be described as the same problem mentioned in Section 2.4. Similarly, for this problem, let $x={\left(x_{1},x_{2},\cdots ,x_{N}\right)}^{T}$ and $y={\left(y_{1},y_{2},\cdots ,y_{N}\right)}^{T}$, where $x_{i},y_{i}\in \mathbb{R}$ is the coordinate value of the *i*th point in $M^{d}$ and ${\mathbb{R}}^{m}$. This problem can also be transformed into finding $y_{i}\in \mathbb{R}$ to minimize $\sum_{i,j}{\left(y_{i}-y_{j}\right)}^{2}W_{ij}$ under reasonable constraints. By the approach below, low-dimensional manifolds of the *N* data points, $y={\left(y_{1},y_{2},\cdots ,y_{N}\right)}^{T}$ can be found; that is, the sequence of eigenvalues of vibration signals after the redundant information was eliminated.

## 3. Experiment Design

**Figure 5:**
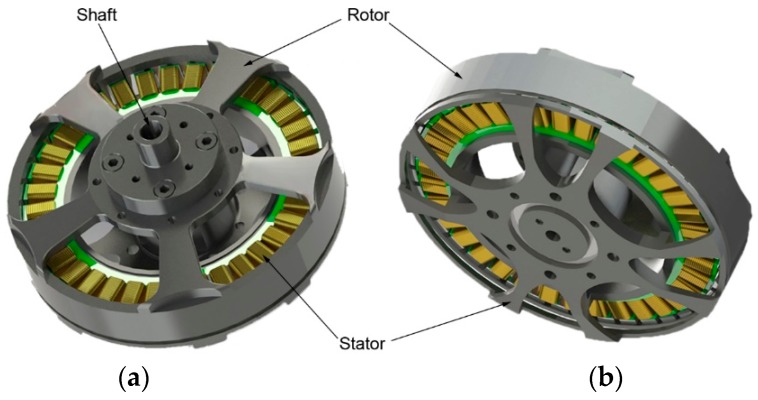
Conceptual graph of the U7 KV170 motor produced by T-MOTOR: (**a**) Front; (**b**) Back.

**Figure 6:**
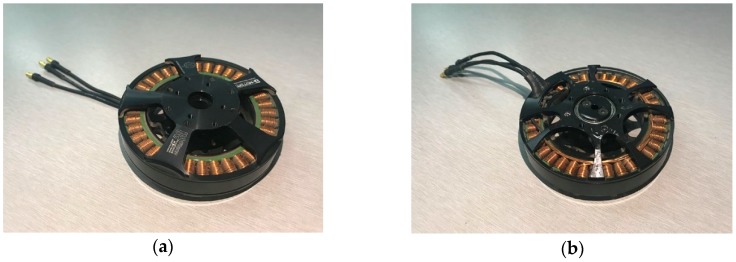
Photos of the U7 KV170 motor used in experiment: (**a**) Front; (**b**) Back.

The motor selected to be tested in this experiment was the U7 KV170 motor produced by T-MOTOR Company in Nanchang, China. Figure 5 shows the conceptual graph of the U7 KV170 motor, and Figure 6 shows photos of the tested motor. It is mainly used in industrial and commercial fields. As a disc-type BLDC motor, it is controlled by an electronic governor and is commonly used in the power systems of multi-rotor UAVs. Its basic parameters are listed in Table 1.

**Figure 7:**
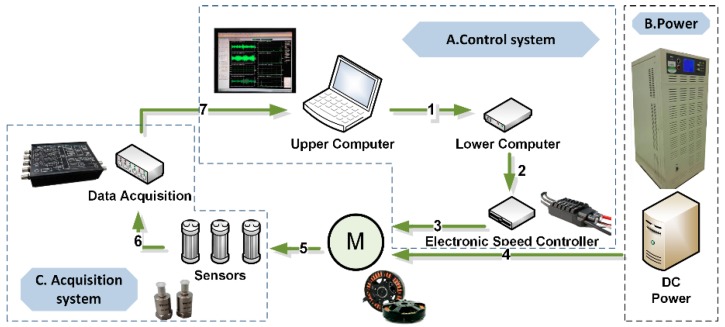
Experimental setup.

**Figure 8:**
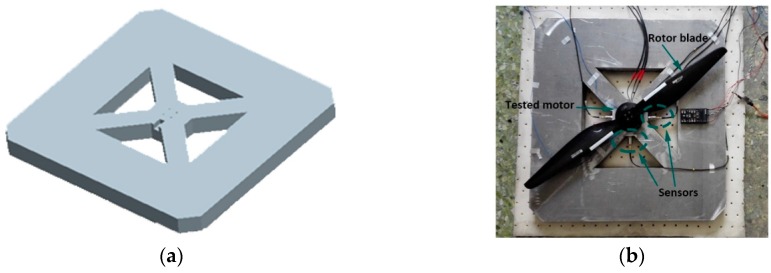
Fixture and devices used in the experiment: (**a**) fixture; (**b**) experiment devices.

Figure 7 demonstrates the experimental setup for vibration signal collection, and the numbers represents the connection between the equipment. The experiment system consists of three subsystems, including A: control system, B: power, and C: acquisition system. In the control system, to ensure the stable operation of the motor, the control connection mode of the upper computer–lower computer–electronic speed controller was used to control the motor’s running state. Numbers 1–3 represent the control connection in the control system. As for power supply, the battery used in regular UAVs was replaced with constant voltage DC (direct current) power to guarantee that the power supply would not affect the working state of the motor, and to avoid the influence of the change of the working environment on the data acquisition as much as possible. This connection is expressed by connection No. 4 in Figure 7. The acquisition system captures vibration signals by connection No. 5 between sensors and the motor. In virtue of analog-to-digital conversion equipment, the vibration signals are uploaded to the upper computer (connection No. 6–7). The specific functions are listed in Table 2. Moreover, in order to effectively avoid the vibration noise caused by the interaction between the external environment and the tested equipment, it was also necessary to effectively isolate the tested equipment (as shown in Figure 8). To simulate the vibration signals collected by the UAV under normal working conditions, the tested motor was installed with a carbon paddle to collect the running signals of the motor with load. The specific parameters of the motor and paddle are shown in Table 3.

For a general rotating mechanical part, its radial signals are less stable than its axial signals. Since its radial signals are more susceptible to mechanical damage, they are more sensitive to faults. Therefore, in this experiment, the radial signals of the tested motor were mainly collected and analyzed, whereas the axial signals were collected to assist in observing the working state of the motor and the tested device.

**Figure 9:**
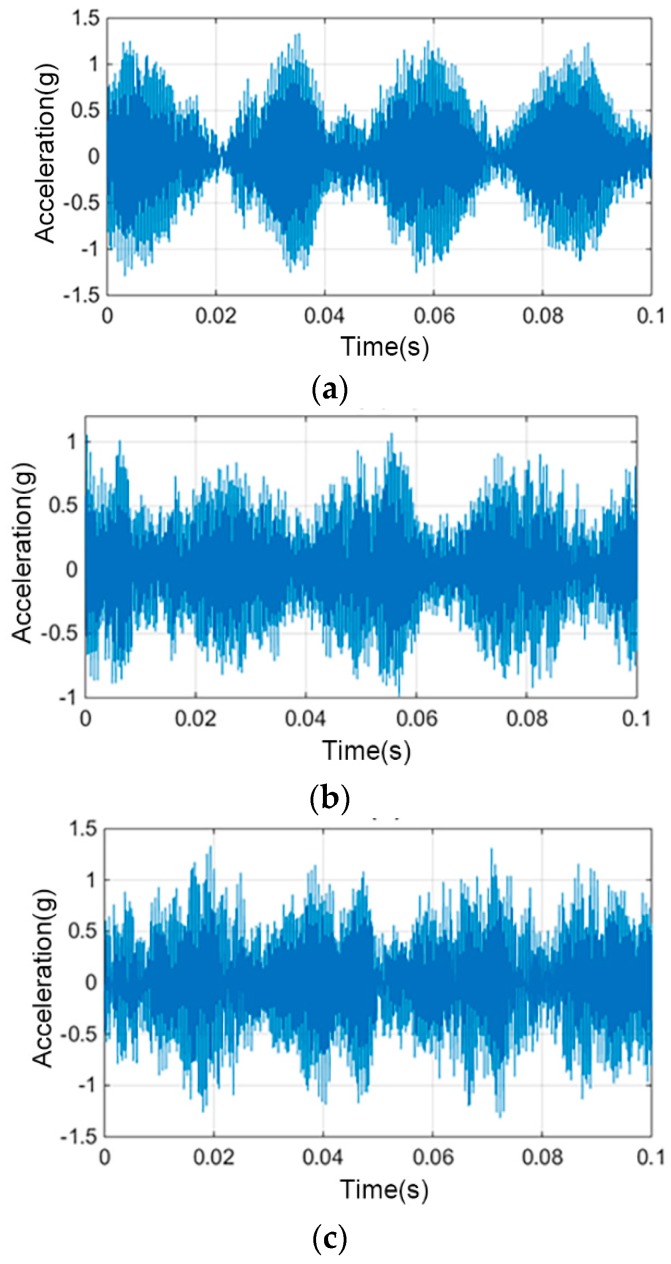
Comparison of time-domain signals after operating for 7.5 h (**a**), 975 h (**b**), 1020 h (**c**).

In this experiment, the motor had obviously abnormal rotation, and could not work normally after running for 975 h. To collect the operational data of the motor after the fault, after the 975th hour, we continued the experiment for 1062 h to collect the vibration signals in the fault state, and to compare them with the vibration signals collected in the normal working state. Figure 9 shows the time-domain vibration signals collected after the motor had been running for 7.5 h, 975 h, and 1020 h (fault state). It can be clearly found through comparison that there was a significant difference in the vibration signals of the motor before and after the operation, as well as before and after the fault.

## 4. Data Processing and Analysis

### 4.1. Data Processing

Unlike fault diagnosis, which usually selects signal characteristics that show sudden changes between the normal and fault conditions, predicting the state of health based on the abrasion process requires signal characteristics that show gradual changes in the abrasion process. The selected vibration signal characteristic value should have a clear trend of change with the life loss, which is different from the characteristic value in general fault diagnosis. Therefore, a variety of characteristics of the vibration signals collected in the experiment were calculated, in order to select the appropriate eigenvalues. In addition, considering the complexity of the high-frequency noise components, the eigenvalues of vibration signals were calculated in the full-frequency and low-frequency bands.

**Figure 10:**
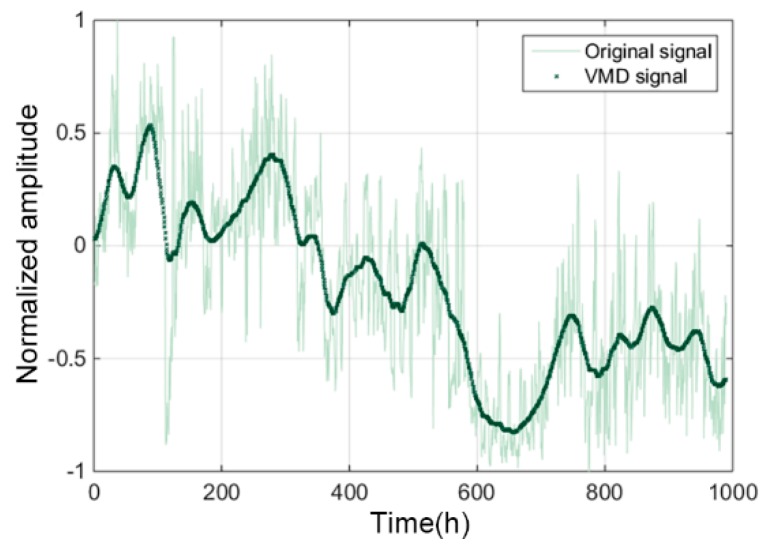
Effect of VMD in decomposing and extracting characteristics trends.

**Figure 11:**
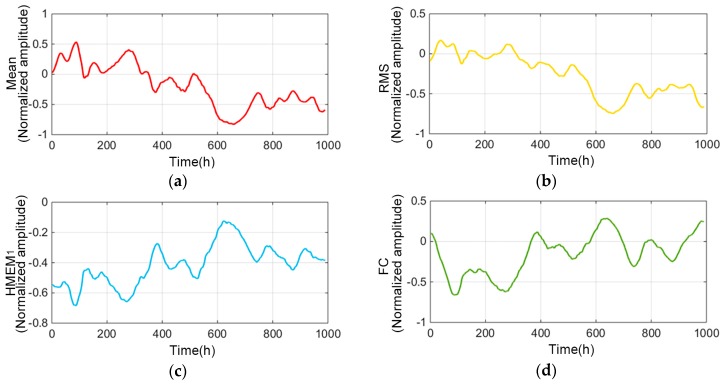
Parts of trend characteristics: (**a**) Mean, (**b**) RMS, (**c**) HMEM_1_, (**d**) FC.

The example in Figure 10 shows a trend curve before and after VMD decomposition extraction. The VMD method can effectively decompose the high and low frequency components in the original vibration signals, thereby selecting the gradual degradation components related to the running time. Through decomposition and screening, the characteristic trends of components that can effectively characterize the degradation process was extracted. Four typical degradation trends are demonstrated in Figure 11 (The specific abbreviations of characteristics are listed in Table A1 of Appendix A).

**Figure 12:**
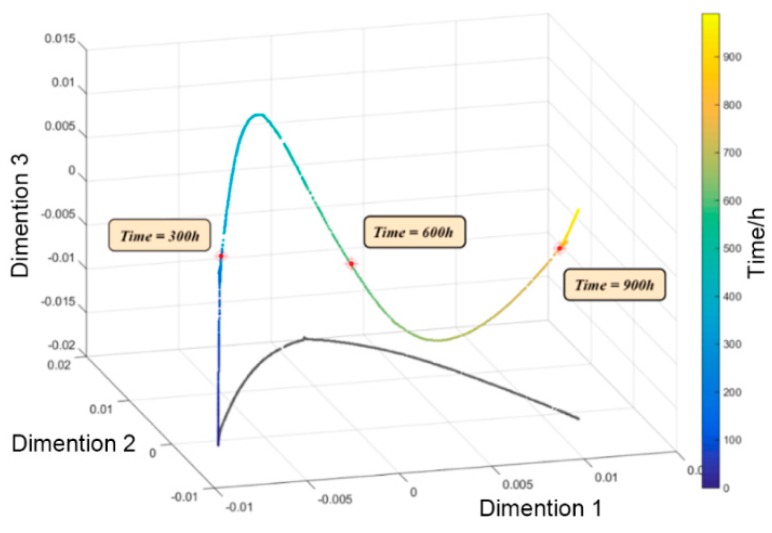
Trends of degradation characteristics after manifold dimension reduction using the method.

Based on the 18 dimensions of the special trend time series obtained by the VMD method, a manifold description of the multi-dimensional degradation characteristics of the motor was established, according to the degradation characteristics extraction method proposed in the previous section. The purpose of the description was to achieve information extraction and dimension reduction of the 18-dimensional data characteristics. According to the LE method, the time series of the original characteristics trend of the motor exists in the space ${\mathbb{R}}^{m}$, where m=18, and the characteristics described in this space are from the original degradation process collected in this experiment without effective information extraction. However, due to the large amount of duplicate information and information that is irrelevant to degradation in the original data, the degradation characteristics of the data can be well-described by the space $M^{d}(d<m)$. Therefore, the mapping relationship from ${\mathbb{R}}^{m}$ to $M^{d}$, $g=f^{-1}$, was constructed. According to this mapping relationship, the degradation process of the motor in the space $M^{d}(d=3)$ was obtained, as shown in Figure 12.

### 4.2. Analysis and Comparison

**Figure 13:**
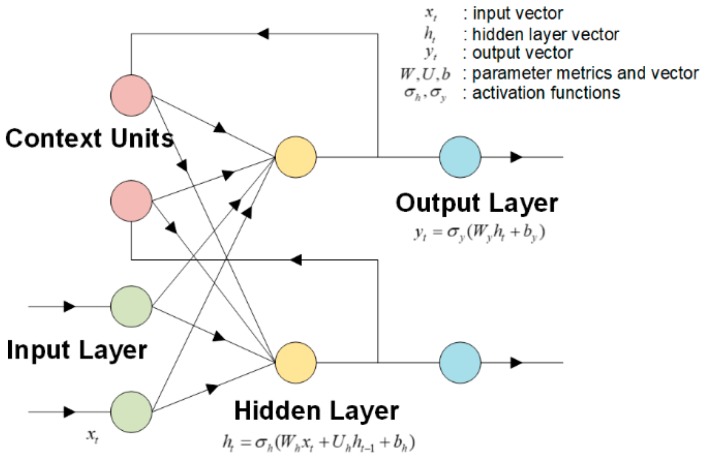
Principle of the Elman neural network.

To verify the effectiveness of the characteristics extraction method proposed in this study, the commonly used Elman neural network prediction method was used to compare the original characteristics signals and the processed characteristics signals. As a typical local regression network, the Elman neural network has certain delayed memory ability and stable performance. It is often used for time series prediction. Figure 13 shows the structural principle of the Elman neural network.

For the purpose of comparison, the data of the degradation characteristics of the total life m of the motor was divided into two categories. The data collected in the early stage, $S_{train}\left(s_{1},s_{2},\cdots ,s_{n}\right),n=945$, was used as the training data of the neural network. The characteristics prediction data of the latter 45 h, ${\hat{S}}_{test}\left({\hat{s}}_{946},{\hat{s}}_{947},\cdots ,{\hat{s}}_{m}\right),m=990$, was obtained. The actual characteristics data of the final 45 h, $S_{test}\left(s_{946},s_{947},\cdots ,s_{m}\right),m=990$, was used as the comparative data, and the difference between the predicted data, ${\hat{S}}_{test}$, and the actual data, $S_{test}$, was evaluated by the average relative error:

(7) $$\bar{\delta }=\frac{1}{m-n}\sum_{i=n+1}^{m}\delta_{i}=\frac{1}{m-n}\sum_{i=n+1}^{m}\frac{\left|{\hat{s}}_{i}-s_{i}\right|}{\left|s_{i}\right|},$$

**Figure 14:**
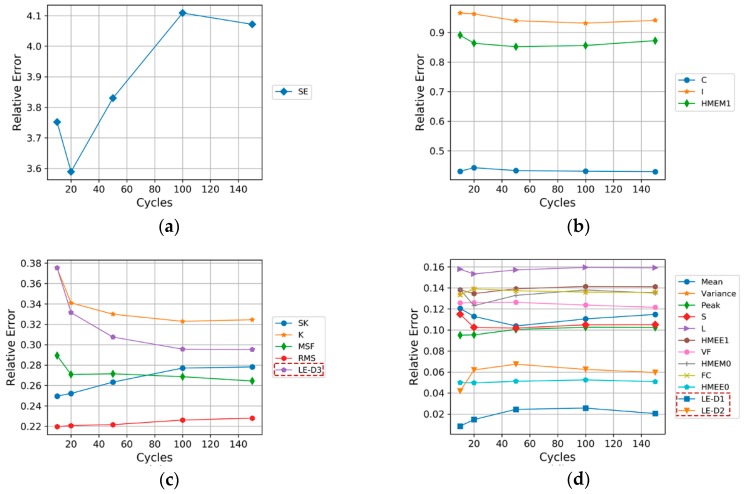
Comparison of prediction errors of characteristics type: (**a**) more than 3.5, (**b**) between 0.4 and 1, (**c**) between 0.2 and 0.4, (**d**) between 0.01 and 0.2.

To avoid the error caused by the construction of a single neural network, the multiple neural network prediction results were averaged in the comparison to obtain representative relative errors. We calculated the mean of prediction error for 20–140 cycles. The relative errors of all features by prediction 20–140 times is demonstrated in Figure 14. It was observed whether the degradation characteristics trend processed by the VMD and LE methods were beneficial for the prediction of the service life and the working state of the motor.

According to the average relative errors, each original characteristics trend (e.g., SE, C, and SK; the specific characteristic symbols are shown in the Appendix A) and the characteristics trends of the first three dimensions obtained after the dimension reduction (i.e., LE-D1, LE-D2 and LE-D3) were compared successively:

- The relative error of index SE was as high as 360–400% (Figure 14a).
- The relative errors of indexes C, I, and HMEE1 were about 40–100% (Figure 14b).
- The relative error of index LE-D3, which was about 30%, was close to the relative errors of indexes SK, K, MSF, and RMS (Figure 14c).
- The relative errors of indexes LE-D1 and LE-D2 were relatively low at about 2–6% (Figure 14d).

As shown in Figure 14, among the characteristic data whose dimensions were reduced using the LE method (LE-D1, LE-D2, LE-D3), the prediction error of the main component of the first dimension (LE-D1) has obvious advantages compared with other single-characteristics degradation data. Since LE-D1 contains sufficient degradation trend information, a low and stable prediction error can be maintained when the same prediction method is used. Nevertheless, as the number of dimensions increases, the effectiveness of the secondary dimension of the LE method will gradually decline. Importantly, the degradation characteristics data obtained by this method is more suitable for the evaluation and prediction of the degradation process of the motor in the working state, and it contains significantly less redundant information that can interfere with subsequent prediction and evaluation than the unprocessed original degradation characteristics data.

## 5. Conclusions

Because of complex background noises and weak distinction, it is hard to directly identify degradation fault signals of the multi-rotor UAV motor during its operation with load. To solve this problem, a weak degradation characteristics extraction method based on the basic principles of VMD and LE was proposed in this study to accurately identify the degradation characteristics evolution information before the fault, and to strengthen the predictability of weak degradation fault characteristics. In this study, with multiple types of basic characteristics of vibration signals used as the basic input, on the basis that the background noises of time series signals were removed using the VMD method, dimension reduction was conducted on the intrinsic information of multi-dimensional characteristics by manifold learning. The aim of this method was to obtain the degradation process trend related to information about the motor’s lifespan, and thereby achieve accurate screening of effective information.

As declared in the literature review, the present research mainly focuses on the improvement of signal processing and fault detection of rotating machinery, ignoring the information collection of degradation before failures. Based on information in weak vibration signals, this method can capture degradation trend signals for better estimation of the health status of rotating machinery. In this way, the proposed method extract informative features in weak vibration signals from a variety aspects. Therefore, the degradation trend is acquired clearly. By comparing the prediction methods, it was found that the method proposed in this study can increase the accuracy and stability for predicting the degradation characteristics trend after information screening and dimension reduction, which obviously outperforms the approach of pre-judging the working state based on a single vibration characteristic. Thus, the proposed method is conducive to predicting the lifespan and monitoring the health status of the BLDC motor of UAVs under actual working conditions, so as to help to optimize maintenance strategies, reduce maintenance costs, and improve the working efficiency. However, sufficient data at the failure threshold is still needed to more accurately predict the time of the fault and the optimal time for preventive maintenance. The methods for accurately evaluating the health status of the motor, using limited failure and life expectancy data, remains to be explored.

## Figures and Tables

**Table 1:** Design parameters of the T-MOTOR U7 KV170 motor.

**Resistance**	89 mΩ	**Slot pole**	36N42P
**Shaft Diameter**	15 mm	**Motor size**	Ø86.8 × 26.5 mm
**Weight**	239 g	**No-load current**	1.1 A
**Working Range of the Lithium Battery**	6–12 S	**Maximum power**	528 W

**Table 2:** Connections between equipment.

Sequence Number	Connected Equipment	Functions
**1**	Upper computer and lower computer	Send control command
**2**	Lower computer and electrical speed controller	Send PWM (pulse width modulation) signals
**3**	Electrical speed controller and motor	Current and voltage conversion
**4**	DC (direct current) power and motor	Power supply
**5**	Motor and sensors	Vibration signals acquisition
**6**	Sensors and data acquisition equipment	Send and convert analog signals
**7**	Data acquisition equipment and upper computer	Send digital signals

**Table 3:** Working parameters of the tested motor.

KV Value ^1^	Voltage	Current	Accelerator	Revolving Speed	Sampling Direction	Sampling Frequency	Paddle
170	22.2 V	27 A	100%	2300 rpm	X, Y, Z axial direction	12.8 kHz	28 × 9.2 inch

^1^ The KV value is the characteristic parameter of the BLDC motor; it refers to the revolving speed increased for every 1 V increase in the supply voltage.

## Appendix A

**Table A1:** Symbols of characteristics.

Type of Characteristics	Characteristics	Symbols
Time-domain characteristics	Mean	M
	Variance	Var
	Root-mean-square value	RMS
	Peak	P
	Peak index	C
	Skewness index	SK
	Kurtosis index	K
	Waveform index	S
	Pulse index	I
	Margin index	L
Frequency-domain characteristics	Gravity frequency	FC
	Mean square frequency	MSF
	Frequency variance	VF
Mean of energy spectrum	Marginal energy spectrum mean value (low frequency)	HMEM_1_
	Marginal energy spectrum mean (full frequency domain)	HMEM_0_
Spectral entropy	Amplitude spectrum entropy	SE
	Marginal energy spectrum entropy (low frequency)	HMEE_1_
	Marginal energy spectrum entropy (full frequency domain)	HMEE_0_
